# No association of vitamin D metabolism-related polymorphisms and melanoma risk as well as melanoma prognosis: a case–control study

**DOI:** 10.1007/s00403-012-1243-3

**Published:** 2012-05-11

**Authors:** Annika Schäfer, Steffen Emmert, Jochen Kruppa, Steffen Schubert, Mladen Tzvetkov, Rotraut Mössner, Kristian Reich, Carola Berking, Matthias Volkenandt, Claudia Pföhler, Michael P. Schön, Thomas Vogt, Inke R. König, Jörg Reichrath

**Affiliations:** 1 Department of Dermatology, Venereology, and Allergology, Georg August University Göttingen, Robert-Koch-Strasse 40, 37075 Göttingen, Germany; 2 Institute of Medical Biometry and Statistics, University Lübeck, University Hospital Schleswig–Holstein, Campus Lübeck, Maria-Goeppert-Straße 1, 23562 Lübeck, Germany; 3 Department of Clinical Pharmacology, Georg August University Göttingen, Robert-Koch-Strasse 40, 37075 Göttingen, Germany; 4 Dermatologikum Hamburg, Stephansplatz 5, 20354 Hamburg, Germany; 5 Department of Dermatology, Ludwig-Maximilians-University of Munich, Frauenlobstrasse 9-11, 80337 Munich, Germany; 6 Department of Dermatology, Venereology, and Allergology, University Clinic Saarland, Building 18, 66041 Homburg, Germany

**Keywords:** Melanoma, Vitamin D, Vitamin D receptor (VDR), 25-hydroxyvitamin D-1α-hydroxylase (CYP27B1), 1,25-dihydroxyvitamin D-24-hydroxylase (CYP24A1), Vitamin D binding protein (VDBP), Gene polymorphism

## Abstract

Melanoma is one of the most aggressive human cancers. The vitamin D system contributes to the pathogenesis and prognosis of malignancies including cutaneous melanoma. An expression of the vitamin D receptor (VDR) and an anti-proliferative effect of vitamin D in melanocytes and melanoma cells have been shown in vitro. Studies examining associations of polymorphisms in genes coding for vitamin D metabolism-related proteins (1α-hydroxylase [*CYP27B1*], 1,25(OH)_2_D-24hydroxylase [*CYP24A1*], vitamin D-binding protein [*VDBP*]) and cancer risk are scarce, especially with respect to melanoma. Mainly VDR polymorphisms regarding melanoma risk and prognosis were examined although other vitamin D metabolism-related genes may also be crucial. In our hospital-based case–control study including 305 melanoma patients and 370 healthy controls single nucleotide polymorphisms in the genes *CYP27B1* (rs4646536), *CYP24A1* (rs927650), *VDBP* (rs1155563, rs7041), and *VDR* (rs757343, rs731236, rs2107301, rs7975232) were analyzed for their association with melanoma risk and prognosis. Except *VDR* rs731236 and *VDR* rs2107301, the other six polymorphisms have not been analyzed regarding melanoma before. To further improve the prevention as well as the treatment of melanoma, it is important to identify further genetic markers for melanoma risk as well as prognosis in addition to the crude phenotypic, demographic, and environmental markers used in the clinic today. A panel of genetic risk markers could help to better identify individuals at risk for melanoma development or worse prognosis. We, however, found that none of the polymorphisms tested was associated with melanoma risk as well as prognosis in logistic and linear regression models in our study population.

## Introduction

Cutaneous melanoma represents one of the most aggressive and treatment-resistant human cancers. The increase of the annual incidence rate varies between populations. For white-skinned Caucasian populations, it has been reported in the order of 3–7 % per year [13]. In Europe, the highest incidence rates have been reported in Scandinavia (occurrence of 15 cases per 100 000 inhabitants per year) and the lowest in the Mediterranean countries (occurrence of 5–7 cases per 100,000 inhabitants per year) [18]. The risk for melanoma development increases with age [19] and other demographic, phenotypic, and environmental risk factors such as sunlight exposure [3, 28], particularly an intermittent exposure [19], family history of melanoma, dysplastic nevi, number of nevi, freckling, fair hair, eye and skin color [5]. Breslow tumor thickness at presentation remains the most important single prognostic factor for patients with cutaneous melanoma [36]. The risk for melanoma metastases and, thus, reduced survival increases with increasing tumor thickness. Early detection of thin melanomas is mandatory for a cure. The 5-year survival rate is ~93 % for melanomas with a Breslow tumor thickness <1.5 mm. The survival rate for melanomas thicker than 3.5 mm is ~37 % [35]. Interestingly, independent of any tumor thickness about one-third of the patients never develop melanoma metastases.

Single nucleotide polymorphisms (SNP) associated with melanoma susceptibility or the course of disease have been described as molecular risk factors in genes involved in the regulation of skin pigmentation, such as the *melanocortin-1 receptor* gene [38], in DNA repair, such as the *xeroderma pigmentosum group C* gene [9], in the detoxification of oxidative stress metabolites, such as the *glutathione S*-*transferase* gene [29], in the regulation of the immune system, such as the *interleukin*-*10* gene [48], or in the production of proteins of the steroid and thyroid hormone superfamily, including the peroxisome proliferator-activated receptor genes [39].

The vitamin D endocrine system regulates a broad spectrum of independent biological processes including innate immune responses, bone metabolism, and cell proliferation and differentiation [23]. Vitamin D, absorbed from the diet or synthesized in the skin by the action of sunlight, is metabolized in the liver to 25-hydroxyvitamin D [25(OH)D] and afterwards in the kidney to 1,25-dihydroxyvitamin D [1,25(OH)_2_D]. There are two important enzymes involved in the formation of circulating active vitamin D (1,25(OH)_2_D_3_ or calcitriol): The hepatic mitochondrial vitamin D 25-hydroxylase (25OHase, CYP27A1) and the renal mitochondrial enzyme 1α-hydroxylase (1αOHase, CYP27B1) for 25(OH)D_3_ and 1,25(OH)_2_D_3_, respectively. The 1,25(OH)_2_D-24-hydroxylase (24OHase, CYP24A1) enzyme plays a critical role in the metabolism of 1,25(OH)_2_D_3_ [24, 40]. The active form of vitamin D (1,25(OH)_2_D_3_ or calcitriol) exhibits a potent secosteroid hormone that binds to a corresponding intranuclear receptor (VDR) in target tissues and thereby mediates its different regulatory transcriptional activities [6, 53]. The vitamin D-binding protein (VDBP) may also play a role in the vitamin D metabolism, because the protein regulates the circulating level of vitamin D [4, 52].

A contribution of the vitamin D system to the pathogenesis and prognosis of malignancies including cutaneous melanoma has been reported [42]. In general 1,25(OH)_2_D_3_, the hormonal derivative of vitamin D_3_ and the ligand of the VDR, exhibits anti-proliferative and pro-differentiation effects in VDR-expressing cell types [16, 37, 43, 49]. It has been shown that melanocytes as well as melanoma cells express VDR and that 1,25(OH)_2_D_3_ has an anti-proliferative effect on melanocytes as well as melanoma cells in vitro [11, 45]. Randerson-Moor et al. [44] showed that that serum 25-hydroxyvitamin D(3) levels were inversely correlated with melanoma Breslow tumor thickness supporting the view that vitamin D and VDR may influence melanoma susceptibility, and putatively melanoma progression even to a greater extend.

The analysis of polymorphisms in genes of the vitamin D metabolism concerning their association with melanoma as well as other types of cancer is a current research topic. For example, Halsall et al. [22] assessed the association of a novel adenine–guanine substitution 1,012 bp upstream of the exon 1a transcription start site (A-1012G) in the *VDR* gene with melanoma risk and prognosis in 171 melanoma patients and 80 healthy controls. The authors found that the A allele was significantly associated with melanoma susceptibility as well as the development of melanoma metastases. Santonocito et al. [47] reported a correlation of the VDR SNP BsmI (rs1544410) with an increased melanoma risk as well as a strong association with an increased Breslow tumor thickness. In another study, the Taq I (rs731236) and the Fok I (rs2228570) SNPs of the *VDR* gene were analyzed for their melanoma risk association. The Tt and the tt genotypes of the VDR-Taq I-polymorphism showed an association with a decreased melanoma risk compared to the TT genotype, whereas the VDR-Fok I Ff genotype was associated with an increased risk to develop melanoma compared to the FF genotype [34]. In a recent analysis, Orlow et al. analyzed 38 common VDR gene polymorphisms in relation to melanoma risk in a large population-based case control study comprising 3,676 individuals with incident primary melanoma [41]. The authors report modest but statistically significant associations between eight SNPs and the risk of developing subsequent new primary melanomas. These results support the hypothesis that the vitamin D pathway plays an important role in the genesis of melanoma [41].

In most studies that investigated the association of polymorphisms in the vitamin D system and skin cancer risk *VDR* gene SNPs were assessed. Information about SNPs in other vitamin D metabolism-related genes that might contribute to skin cancer or other types of internal cancers is scarce. Flohil et al. [15] analyzed the SNPs rs7041 and rs4588 in the *VDBP* gene for their association with basal cell carcinoma (BCC). Only the AT variant of rs7041 was associated with an increased BCC risk in younger patients after age stratification. The minor allele of the *CYP24A1* gene SNP rs927650 has been reported to be associated with a decreased risk of disease recurrence/regression in prostate cancer patients [26]. The *CYP27B1* single nucleotide polymorphism rs4646536 was not associated with the susceptibility for developing colon cancer [14].

In our present hospital-based case–control study, we tested eight vitamin D metabolism-related polymorphisms focusing on the *VDBP* gene (rs1155563 and rs7041) and the genes coding for *CYP27B1* and *CYP24A1* (rs4646536 and rs927650) for their association with melanoma risk and melanoma prognosis. In addition, four SNPs in the *VDR* gene (rs2107301, rs7975232, rs757343, and rs731236) were assessed in our 305 melanoma patients and 370 healthy controls study group.

## Materials and methods

### Study subjects

We analyzed a total study group of 675 probands. The study group consisted of 305 patients with histopathologically confirmed cutaneous melanoma and 370 healthy control donors from the same area (gender and ethnicity matched). The recruitment period was from 2001 to 2003 in the Departments of Dermatology of the Ludwig-Maximilians-University Munich and the Georg-August-University Goettingen. Control probands were recruited from the donors at blood transfusions services from the same hospitals as well as from local health care personnel known personally by the authors. After completion of personal interviews, blood samples were obtained from each proband and the genomic DNA was extracted and stored. The following parameters from patients and controls were registered by standardized procedures: sex, age, hair color, eye color, skin type, number of nevi (on both forearms, diameter >2 mm), primary tumor thickness. These studies were in accordance with the national protocols approved by the Georg-August-University and Ludwig-Maximilians-University Institutional Review Boards. Informed consent was obtained from all the study participants orally as well as in writing.

### Genotyping

The following SNPs were analyzed with pre-designed genotyping assays: rs731236 (*VDR*), rs7975232 (*VDR*), rs2107301 (*VDR*), rs757343 (*VDR*), rs4646536 (*CYP27B1*), rs927650 (*CYP24A1*), rs7041 (*VDBP*) (assay numbers C_2404008_10, C_28977635_10, C_16174096_10, C_2404009_20, C_25623453_10, C_7595497_10, C_3133594_30, respectively). The SNP rs1155563 (*VDBP*) was analyzed using a Custom Taqman SNP Genotyping Assay. All ready-to-use TaqMan SNP Genotyping assays (40× concentrated) and TaqMan Genotyping Mastermix (2× concentrated) were purchased from Applied Biosystems (Foster City, USA) and applied according to the manufacturer’s instructions. PCR amplification was carried out in 384 well plates (Frame Star 384, purple frame, Thermo Fisher, Waltham, USA) in an Eppendorf 384-well Mastercycler (Eppendorf, Hamburg, Germany). Reactions were performed in a total volume of 5 μl including 10 ng genomic DNA. Genotyping was done using a Taqman 7200 HT (Applied Biosystems, Foster City, USA). For control purposes, the sex was genetically re-evaluated using a pre-designed Taqman genotyping assay (AMELXY) as described previously [54]. The genetically determined sex was discordant in 8 probands (of 683 tested) that were subsequently excluded from the analysis. For further control, a minimum of 10 % of all samples were genotyped in duplicates showing 100 % concordance between the genotype calls.

### Statistics

To determine whether the genotype frequencies conformed to the HWE, the equivalence test proposed by Wellek was used (5 % test level) with ε = 0.1 that tests for conformation with HWE instead of deviation from HWE [56]. For identification of the phenotypic, demographic, and environmental risk factors relevant in the study, logistic regression analyses were carried out to predict melanoma from age, nevus count, and skin type as continuous covariates, as well as from gender, hair colour (red vs. other) and eye colour (green or blue vs. grey or brown) as binary covariates. A backward selection by Akaike Information Criterion (AIC) in a stepwise algorithm was conducted. Therefore, the covariates age, nevus count, and skin type were selected for the logistic regression analyses. To explore the effect of single polymorphisms, these were added to an additive and dominant logistic regression model with adjustment by the selected covariates (Tables 3, 4). For associations with the Breslow tumor thickness and log transformed Breslow tumor thickness additive and dominant linear regression models were estimated including the selected covariates (Tables 5, 6, 7 and 8).

Based on the frequencies of the homozygous normal genotype observed in our controls, we estimated the power to detect an OR of 1.5 at a significance level of 5 % in a dominant model. The resulting power exceeded 65 % for all but three SNPs. For these, the estimated power was 36 % (rs2107301), 48 % (rs7041), and 47 % (rs757343).

## Results

### Study subjects

The analysis included 305 unrelated Caucasian patients with histopathologically confirmed cutaneous melanoma and 370 unrelated Caucasian, healthy, cancer-free controls from the same area. All probands were interviewed in standardized procedures to register the following demographic and phenotypic parameters: gender (1: male, 2: female), age, hair color (1: red, 2: blonde, 3: brown, 4: black), eye color (1: green, 2: blue, 3: grey, 4: brown), skin type (I, II, III, IV), number of nevi (on both forearms, diameter >2 mm), and primary Breslow tumor thickness. The male:female distribution was 52.1:47.9 % in the melanoma patients and 52.4:47.6 % in the controls. The mean/median age in the melanoma group was 53.05/55 years and 36.76/34 years in the control group.

Applying logistic regression analysis, there were no independent associations of gender as well as hair color or eye color with the susceptibility to develop melanoma in the study population as indicated in Table 1 (odds ratios (OR) of 1.09, 0.95, 1.12, respectively). A positive family history of melanoma was too rare to analyze. However, the frequencies of the phenotypic and demographic melanoma risk factors skin type (OR: 0.44), number of nevi (OR: 1.04), and age (OR: 1.09) were different in the melanoma patient group compared with the control group. A higher number of nevi was associated with increased melanoma risk. Fair skin type and older age were also independent melanoma risk factors (Table 1). This indicates that our study population is representative of the German population as described earlier [9, 10, 48].

**Table 1:** Association of demographic and phenotypic markers with melanoma risk (280 cases and 337 controls)

Risk factors	OR	95 % CI	*p* value
Gender^b^	1.09	0.72–1.63	0.67
Age^a^	1.09	1.07–1.11	10^−4^
Skin type^a^	0.44	0.33–0.59	10^−4^
Hair color^b^	0.95	0.69–1.30	0.74
Eye color^b^	1.12	0.91–1.36	0.30
Number of nevi^a^	1.04	1.03–1.05	10^−4^

^a^Continuous covariates including skin type I–IV (by increase)

^b^Gender, hair colour (red vs. other) and eye colour (green or blue vs. grey or brown) as binary covariates

### Distribution of the polymorphisms

All polymorphisms analyzed in this study have already been described previously. The SNPs rs4646536 and rs927650 in *CYP27B1* and *CYP24A1*, respectively, are both located in intron regions as is the rs1155563 VDBP SNP. The rs7041 VDBP SNP (Glu432Asp) is located in exon 11. Three of the VDR SNPs are located in intron regions: rs757343, rs2107301, and rs7975232. The synonymous VDR SNP rs731236 is located in exon 9.

Table 2 depicts general information of the eight single nucleotide polymorphisms investigated concerning allele frequencies and genotype distributions. All polymorphisms are quite common. The minor allele frequencies ranged from 0.11 to 0.49. The genotype frequencies of the polymorphisms rs731236, rs7975232, rs7041, rs4646536, rs927650, rs1155563 conformed to the Hardy–Weinberg equilibrium (HWE). The SNPs rs2107301 and rs757343 showed a small deviance from HWE, however, could be incorporated into the further analysis as we performed several control steps. First, we re-analysed about 10 % of all samples and found no deviations. Second, we re-assessed the gender by a pre-designed TaqMan assay (AMELXY).

**Table 2:** Genotype distribution, Hardy–Weinberg equilibrium, and allele frequencies

SNP	Gene	Geno-type	hwe	A1A1	A1A2	A2A2	Minor allele frequency	Minor allele frequency (HapMap2)
rs4646536	CYP27B1	AG	Yes	254	291	72	0.35	0.34
rs927650	CYP24A1	CT	Yes	182	314	120	0.45	0.43
rs7041	VDBP	AC	Yes	105	306	206	0.42	0.43
rs1155563	VDBP	GA	Yes	315	248	54	0.29	0.29
rs731236	VDR	AG	Yes	236	281	100	0.39	0.44
rs757343^a^	VDR	CT	No	487	126	4	0.11	0.12
rs2107301^a^	VDR	AG	No	59	227	331	0.28	0.30
rs7975232	VDR	AC	Yes	165	299	153	0.49	0.43

^a^SNPs were analyzed despite a small deviance of HWE due to internal controls

### Polymorphisms and melanoma risk

Neither of the eight polymorphisms involved in the vitamin D metabolism was found to be associated with the risk to develop cutaneous melanoma in our study population applying logistic regression models with age, skin type, and number of nevi as covariables. This was true for the dominant model (Table 3) as well as the additive genetic model (Table 4). With the dominant model, the CYP27B1 rs4646536 as well as the VDR rs757343 and rs 2107301 SNPs exhibited the lowest *p* values of 0.42, 0.65, and 0.56, respectively, corresponding to ORs of 1.19, 1.12, and 1.22. Based on the confidence intervals, we can exclude true ORs for the dominant genotype of greater than about two for seven out of the eight SNPs with a probability of 95 %. With the additive model, the lowest *p* values were found for the VDBP SNPs rs1155563 and rs7041 as well as for the VDR rs2107301 and again for the CYP27B1 rs4646536 corresponding to 0.16 (OR: 0.80), 0.44 (OR: 0.89), 0.50 (OR: 0.90), and 0.58 (OR: 1.09), respectively. With a probability of 95 %, we can exclude that the true OR from the additive genetic model is greater than 1.5 for seven out of the eight SNPs.

**Table 3:** Association with melanoma risk: results of the dominant logistic regression model including the demographic and phenotypic melanoma risk factors age, skin type, and number of nevi as covariables (280 cases and 337 controls)

SNP	OR	95 % CI	*p* value
rs4646536 (CYP27B1)	1.19	0.79–1.79	0.42
rs927650 (CYP24A1)	0.98	0.62–1.53	0.92
rs7041 (VDBP)	1.01	0.58–1.74	0.99
rs1155563 (VDBP)	0.96	0.45–2.04	0.92
rs731236 (VDR)	0.99	0.65–1.50	0.96
rs757343 (VDR)	1.12	0.69–1.83	0.65
rs2107301 (VDR)	1.22	0.62–2.39	0.56
rs7975232 (VDR)	1.00	0.63–1.58	1.00

**Table 4:** Association with melanoma risk: results of the additive logistic regression model including the demographic and phenotypic melanoma risk factors age, skin type, and number of nevi as covariables (280 cases and 337 controls)

SNP	OR	95 % CI	*p* value
rs4646536 (CYP27B1)	1.09	0.80–1.48	0.58
rs927650 (CYP24A1)	0.99	0.74–1.33	0.95
rs7041 (VDBP)	0.89	0.66–1.20	0.44
rs1155563 (VDBP)	0.80	0.58–1.10	0.16
rs731236 (VDR)	0.96	0.72–1.28	0.79
rs757343 (VDR)	1.09	0.68–1.74	0.73
rs2107301 (VDR)	0.90	0.67–1.22	0.50
rs7975232 (VDR)	1.00	0.75–1.32	0.98

### Polymorphisms and melanoma prognosis

It is well known that an increased Breslow tumor thickness is associated with an increased risk for melanoma metastases and, thus, reduced survival. To explore a potential effect of the SNPs on the risk to develop melanoma metastases a dominant as well as an additive linear regression model was estimated incorporating the Breslow tumor thickness as the dependent variable. Applying both genetic models, the dominant (Table 5) and the additive model (Table 6), no association of any of the eight polymorphisms with the Breslow tumor thickness could be detected. With the dominant model, the *p* values ranged from 0.15 (OR: 0.53) for the VDBP rs1155563 SNP to 0.93 (OR: −0.02) for the CYP27B1 rs4646536 SNP. With the additive model, the lowest *p* values were observed for the VDBP SNPs rs7041 and rs1155563 as well as for the VDR SNPs rs2107301, rs757343, and rs731236 corresponding to 0.14 (OR: 0.22), 0.17 (OR: 0.22), 0.15 (OR: 0.22), 0.41 (OR: −0.19), and 0.43 (OR: 0.12), respectively. Log transformation of the Breslow tumor thickness neither changed the results of the dominant nor the additive linear regression model (Tables 7, 8). Unfortunately, further data on relapse free survival, or over all melanoma specific survival are not documented in our database.

**Table 5:** Association with Breslow tumor thickness (melanoma prognosis): results of the dominant linear regression model including the demographic and phenotypic melanoma risk factors age, skin type, and number of nevi as covariables (263 cases included)

SNP	OR	95 % CI	*p* value
rs4646536 (CYP27B1)	−0.02	−0.43 to 0.39	0.93
rs927650 (CYP24A1)	0.26	−0.18 to 0.69	0.25
rs7041 (VDBP)	0.28	−0.23 to 0.80	0.28
rs1155563 (VDBP)	0.53	−0.19 to 1.25	0.15
rs731236 (VDR)	0.17	−0.24 to 0.57	0.42
rs757343 (VDR)	−0.20	−0.68 to 0.29	0.43
rs2107301 (VDR)	0.19	−0.49 to 0.87	0.58
rs7975232 (VDR)	0.14	−0.34 to 0.62	0.57

**Table 6:** Association with Breslow tumor thickness (melanoma prognosis): results of the additive linear regression model including the demographic and phenotypic melanoma risk factors age, skin type, and number of nevi as covariables (263 cases included)

SNP	OR	95 % CI	*p* value
rs4646536 (CYP27B1)	0.01	−0.29 to 0.30	0.96
rs927650 (CYP24A1)	0.02	−0.27 to 0.31	0.89
rs7041 (VDBP)	0.22	−0.07 to 0.50	0.14
rs1155563 (VDBP)	0.22	−0.09 to 0.52	0.17
rs731236 (VDR)	0.12	−0.18 to 0.42	0.43
rs757343 (VDR)	−0.19	−0.65 to 0.26	0.41
rs2107301 (VDR)	0.22	−0.08 to 0.52	0.15
rs7975232 (VDR)	−0.01	−0.30 to 0.28	0.95

**Table 7:** Association with log-transformed Breslow tumor thickness (melanoma prognosis): results of the dominant linear regression model including the demographic and phenotypic melanoma risk factors age, skin type, and number of nevi as covariables (263 cases included)

SNP	OR	95 % CI	*p* value
rs4646536 (CYP27B1)	0.03	−0.19 to 0.26	0.77
rs927650 (CYP24A1)	0.01	−0.23 to 0.25	0.93
rs7041 (VDBP)	0.14	−0.13 to 0.42	0.31
rs1155563 (VDBP)	0.29	−0.11 to 0.68	0.15
rs731236 (VDR)	0.16	−0.06 to 0.38	0.15
rs757343 (VDR)	−0.01	−0.27 to 0.26	0.95
rs2107301 (VDR)	−0.03	−0.40 to 0.34	0.86
rs7975232 (VDR)	−0.01	−0.27 to 0.25	0.94

**Table 8:** Association with log-transformed Breslow tumor thickness (melanoma prognosis): results of the additive linear regression model including the demographic and phenotypic melanoma risk factors age, skin type, and number of nevi as covariables (263 cases included)

SNP	OR	95 % CI	*p* value
rs4646536 (CYP27B1)	0.06	−0.10 to 0.22	0.48
rs927650 (CYP24A1)	−0.05	−0.21 to 0.11	0.56
rs7041 (VDBP)	0.09	−0.06 to 0.24	0.25
rs1155563 (VDBP)	0.10	−0.07 to 0.26	0.27
rs731236 (VDR)	0.12	−0.04 to 0.28	0.15
rs757343 (VDR)	−0.02	−0.27 to 0.23	0.88
rs2107301 (VDR)	0.03	−0.13 to 0.20	0.71
rs7975232 (VDR)	−0.07	−0.22 to 0.09	0.39

## Discussion

Among human cancers, the cutaneous melanoma represents a very aggressive and treatment-resistant type of cancer with a steeply increasing annual incidence rate [13]. There is a need to identify further molecular genetic markers for the susceptibility to develop melanoma in addition to the well known, but quite crude, environmental, demographic, and phenotypic markers like sunlight exposure [3, 28], family history of melanoma, dysplastic nevi, number of nevi, freckling, fair hair, eye and skin color [5] as well as for a better estimation of melanoma prognosis [36].

Generally, the vitamin D system regulates different biological processes. It is involved in the regulation of cell proliferation and differentiation [23]. The major enzymes involved in generating the active form of vitamin D are the hepatic mitochondrial vitamin D-25-hydroxylase (25OHase, CYP27A1) and the renal mitochondrial enzyme 1α-hydroxylase (1aOHase, CYP27B1) [24, 40]. Once activated, the secosteroid hormone binds to the VDR receptor. The complex is then internalized and functions as a transcription factor [6, 53]. While 1,25(OH)_2_D-24-hydroxylase (24OHase, CYP24A1) is responsible for the degradation of the activated vitamin D [24, 40], the vitamin D-binding protein regulates the circulating level of active vitamin D [4, 52]. Every single one of these enzymes plays an essential role in the metabolism of vitamin D: Either in the formation of active vitamin D, in its action, or in its degradation. Consequently, every vitamin D-related enzyme is involved directly or indirectly in the outcome of the function of the secosteroid hormone. For melanoma cells as well as melanocytes, an anti-proliferative effect of 1,25(OH)2D3 in vitro has been shown [11, 45]. Reichrath et al. [46] reported that the VDR receptor is expressed in primary melanoma tissue. Furthermore associations between reduced serum levels of 25-hydroxyvitamin D and cancer have been reported [20, 50, 51].

In this present hospital-based case–control study, we analyzed putative associations of 8 SNPs in vitamin D metabolism-related genes with melanoma risk as well as melanoma prognosis in a study group of 305 melanoma patients and 375 controls. Most of the studies examining associations between skin cancer and polymorphisms in the vitamin D metabolism analyzed polymorphisms in the *VDR* gene [27, 33, 34, 47]. Studies about polymorphisms in other genes contributing to the vitamin D status and their possible associations to skin cancer risk are rare. To our knowledge, only the rs7041 polymorphism in *VDBP* gene was reported to be associated with an increased BCC risk [15]. Polymorphisms in the other vitamin D metabolism-related genes may also be relevant concerning cancer susceptibility. Polymorphisms in the vitamin D metabolism-related genes *CYP24A1* and *CYP27B1* were reported to be associated with colon as well as prostate cancer risk, respectively [8, 14].

We analyzed the rs4646536 SNP in the *CYP27B1* gene. This gene seems relevant for carcinogenesis as other polymorphisms in this gene were found to be associated with prostate cancer [26] and colon cancer [14]. The rs4646536 polymorphism has rarely been analyzed with respect to cancer susceptibility before. An examination of this SNP with respect to prostate cancer by Beuten et al. [8] revealed no association. An association of this SNP with melanoma was not investigated before.

Dong et al. [14] concluded that the *CYP27B1* and *CYP24A1* genes as important players in vitamin D metabolism have significance in colon cancer risk. Moreover, associations of SNPs in the *CYP24A1* gene with prostate cancer prognosis have been reported. The polymorphism rs927650 that is analyzed in our present study, as well as another polymorphism (rs2762939) in the *CYP24A1* gene were significantly associated with prostate cancer prognosis [26]. With regard to melanoma and SNP rs927650 no previous data exist.

We also analyzed two SNPs in the *VDBP* gene, rs1155563 and rs7041. These polymorphisms may have a functional consequence. Three common haplotypes (GC1S, GC1F, and GC2) are existent in VDBP that is also referred to as GC protein. The haplotypes differ in the combination of two SNPs including rs7041. These haplotypes have been associated with differences in VDBP and serum 25(OH)D concentration as well as affinity of VDBP to vitamin D metabolites. The GC1S and GC1F haplotypes were associated with higher levels of VDBP and serum 25(OH)D concentration as well as higher affinity of VDBP to vitamin D metabolites compared with the GC2 haplotype [4, 31, 32]. In genome wide association studies of circulating vitamin D levels, the rs7041 and rs1155563 polymorphisms were found to be associated with another common VDBP SNP, rs 2282679. These three common VDBP variants, with the rs2282679 prevailing, conferred an association signal with 25(OH)D levels in metaanalyses [2, 55]. It was recently reported that analysis of HapMap determined that rs1155563 is in high linkage disequilibrium (LD) with rs4588 (LD = 0.83) [25]. The GC2 allele has been associated with decreased postmenopausal breast cancer risk [1]. Furthermore, the rs7041 VDBP SNP was associated with basal cell carcinoma risk [15]. Neither the rs7041 nor the rs1155563 have ever been analyzed for their association with melanoma so far.

Polymorphisms in the *VDR* gene have been analyzed for their associations with prostate cancer [21], breast cancer [12], colorectal adenomas [30] and also for their association with melanomas [27]. However, studies examining the association between VDR SNPs and melanoma risk and prognosis are relatively rare compared to the other cancer types. The two VDR SNPs analyzed in this study, rs7975232, and rs757343, have not been investigated for their association with melanoma yet. The VDR SNPs rs731236 and rs2107301 have been analyzed with respect to melanoma before [7, 17, 27, 33, 34, 41]. Two studies identified a decreased melanoma risk in association with rs731236 [33, 34], but most studies could not find an association of rs731236 with melanoma risk or prognosis [7, 17, 27, 41].

After the identification of age, skin type, and number of nevi as independent demographic and phenotypic melanoma risk factors in our study group [9, 48], we analyzed every single SNP for its association with melanoma risk in a dominant as well as an additive logistic regression model integrating these risk factors. As the primary tumor thickness was documented, we also analyzed the association of every single SNP with melanoma prognosis in a dominant and an additive linear regression model. In summary, none of the analyzed SNPs showed an association with melanoma risk or melanoma prognosis in our representative study group.

Our study has two major drawbacks. One is the younger age of the controls. We are aware that a better match of patients and controls with regard to age would have been desirable. However, our study population is closely gender and ethnicity matched as we recruited all probands at only two university hospitals in Germany. It could be that the younger controls will develop melanoma at an older age and thus account as “false negatives”. However, this effect may be counteracted by the effect that our older melanoma cohort may be enriched for relevant SNPs due to the fact that controls with the same relevant SNPs may have already died from other types of cancers at that age. As pointed out, the vitamin D system-associated polymorphisms may predispose also to other malignancies. In any case, we have controlled for age in our multivariate regression analyses to compensate for that limitation. Further, we are confident that our study population is representative as we already identified DNA repair gene SNPs [9] and interleukin 10 promoter polymorphisms [48] as independent molecular melanoma risk factors with this study population.

The second drawback is the limited power of our study, which is less than 80 % for any SNP for detecting an OR of 1.5. Based on the confidence intervals for the true ORs, we are able to exclude true ORs of greater than 2.0 in the dominant and greater than 1.5 in the additive genetic model for seven out of the eight SNPs, which we view as an indicator that strong effects of the investigated variants can be excluded by our study.

Further studies with larger study populations and an optimized age match of controls seem mandatory.
